# A Review on Microbiological Source Attribution Methods of Human Salmonellosis: From Subtyping to Whole-Genome Sequencing

**DOI:** 10.1089/fpd.2023.0075

**Published:** 2024-03-06

**Authors:** Rebeca Cardim Falcao, Megan R. Edwards, Matt Hurst, Erin Fraser, Michael Otterstatter

**Affiliations:** ^1^ British Columbia Centre for Disease Control, Vancouver, Canada.; ^2^ School of Population and Public Health, The University of British Columbia, Vancouver, Canada.; ^3^ Public Health Agency of Canada, Guelph, Canada.

**Keywords:** source attribution, frequency-matching methods, *Salmonella*, whole-genome sequence, microbiological, machine learning, population genetic methods

## Abstract

is one of the main causes of human foodborne illness. It is endemic worldwide, with different animals and animal-based food products as reservoirs and vehicles of infection. Identifying animal reservoirs and potential transmission pathways of *Salmonella* is essential for prevention and control. There are many approaches for source attribution, each using different statistical models and data streams. Some aim to identify the animal reservoir, while others aim to determine the point at which exposure occurred. With the advance of whole-genome sequencing (WGS) technologies, new source attribution models will greatly benefit from the discriminating power gained with WGS. This review discusses some key source attribution methods and their mathematical and statistical tools. We also highlight recent studies utilizing WGS for source attribution and discuss open questions and challenges in developing new WGS methods. We aim to provide a better understanding of the current state of these methodologies with application to *Salmonella* and other foodborne pathogens that are common sources of illness in the poultry and human sectors.

## Introduction

In studies of human foodborne illnesses, such as those caused by *Salmonella*, *Campylobacter*, and *Escherichia coli*, attributing the source of the pathogen is essential for better understanding transmission dynamics and developing efficient control strategies. Source attribution methods attribute human cases caused by a foodborne disease to different sources (Mughini-Gras et al., 2019; Pires et al., 2009).

They quantify the contribution of each source to the human disease burden through their linkage. This can help with the prioritization of intervention strategies. Source is a broad term, meaning the origin of the pathogen, and includes a range of groups, such as animal reservoirs and vehicles, depending on the attribution problem being tackled (Pires et al., 2009). For zoonotic pathogens, like *Salmonella*, animals may be hosts (organisms that harbour the pathogen) or carriers (hosts without discernible illness), where the pathogen lives and multiplies and are known as animal reservoirs. The transmission vehicles represent ways pathogens can travel from the reservoirs to humans.

Food, environment, and direct contact with animals are examples of a vehicle (Mughini-Gras et al., 2019; Pires et al., 2009; Wagenaar et al., 2013). These components can be potential sources in source attribution studies (Carstens et al., 2019; Ferrari et al., 2019). Pires et al. (2009) define points of attribution as “points in the food chain where human illness attribution can take place, such as production, distribution, and consumption.” There are many approaches to source attribution, depending on the goal, questions of the study, data availability, and point of attribution (Mather et al., 2015; Mughini-Gras et al., 2019; Pires et al., 2014). The diversity of potential transmission sources can greatly complicate attempts at attribution. One of the reasons is the need to have robust and representative samples from all true sources (Mather et al., 2015; Mughini-Gras et al., 2019; Pires et al., 2014; Pires et al., 2009).

*Salmonella* is one of the most common causes of foodborne illness in the world (Kirk et al., 2015). *Salmonella* is a genus of Gram-negative rod-shaped bacteria comprising two species: *Salmonella enterica* and *Salmonella bongori*. *Salmonella enterica* is further categorized into six subspecies: *Salmonella enterica* subsp. *enterica*; *Salmonella enterica* subsp. *salamae*; *Salmonella enterica* subsp. *arizonae*; *Salmonella enterica* subsp. *diarizonae*; *Salmonella enterica* subsp. *houtenae*; and *Salmonella enterica* subsp. *indica* (Brenner et al., 2000; Eng et al., 2015). *Salmonella enterica* subsp. *enterica* is responsible for most *Salmonella* infections in humans (Eng et al., 2015; Kirk et al., 2015).

More than 2600 serotypes of *Salmonella* have been identified to date, and at least 50% of these serotypes belong to *Salmonella enterica* subsp. *enterica* (Eng et al., 2015; Thames and Theradiyil Sukumaran, 2020; World Health Organization, 2018). The most frequent Salmonella serovar isolated from reported human cases in Canada was *Salmonella* Enteritidis, corresponding to 35% of cases (Government of Canada, 2020). *Salmonella* outbreaks are frequently linked to animal reservoirs such as chickens (Hoelzer et al., 2011; Wessels et al., 2021) and pigs (Bearson, 2022; Hoelzer et al., 2011), and table eggs (vehicle) (Chousalkar et al., 2018; Popa and Papa, 2021).

In addition, food products such as cheese pasta, infant formula, mayonnaise, cucumbers, and other vegetables have been linked to cases of salmonellosis (Carstens et al., 2019; Laughlin et al., 2019; Popa and Papa, 2021), likely due to environmental or cross-contamination during processing. *Salmonella* can cause illness in hosts ranging from poultry to humans, with clinical manifestations of disease spanning enteric fever, gastroenteritis, bacteremia, and an asymptomatic chronic carrier state (Eng et al., 2015; World Health Organization, 2018). In livestock, including poultry, asymptomatic and persistent infection in the animal's digestive tract result in a carrier state, further facilitating transmission to humans (Hoelzer et al., 2011; Silva et al., 2014). Moreover, rodents, known carriers of *Salmonella*, can contaminate barn and farm environments (Anderson et al., 2006; Hoelzer et al., 2011).

*Salmonella* can also colonize plants in the field (Holden et al., 2009; Jechalke et al., 2019), survive in fresh produce (Beuchat, 2002; Critzer and Doyle, 2010), and survive in soil and water (Jechalke et al., 2019), underscoring the breadth of potential transmission sources (Silva et al., 2014). Understanding the complex interactions between humans, animals and their environments that may lead to disease spread is necessary to identify and address transmission pathways of pathogens such as *Salmonella*. A one health approach using multidisciplinary collaborative efforts is essential to develop effective methods, public policy, and interventions aimed at source attribution and disease control in host populations (Destoumieux-Garzón et al., 2018; Silva et al., 2014).

Source attribution provides several methods for evaluating interventions and pathways of transmission and infection, with varying data sources and data quality requirements. These methodologies include microbiological, epidemiological, expert elicitation, and intervention studies, with the choice of method often driven by the type and quality of available data (Pires et al., 2014; Pires et al., 2009). Aiding in the detection of local and global outbreaks of foodborne diseases is PulseNet, a laboratory network comprising standardized subtyping data of foodborne pathogens around the globe.

Most of its data consist of pulsed-field gel electrophoresis (PFGE) subtyping analysis, although globally and in Canada since 2017, PFGE has been replaced by whole-genome sequencing (WGS) (Government of Canada, 2020). It is noteworthy that, in the poultry industry, a primary source of *Salmonella*, several interventions have been tried to reduce *Salmonella* prevalence, including vaccination, cleaning and sanitation of barns, separation of flocks, and testing, with the aim to lessen human cases (Dórea et al., 2010; Totton et al., 2012; Trampel et al., 2014). Similar interventions have also been used in pigs and cattle (da Costa et al., 2021; Holschbach and Peek, 2018). However, assessing the efficacy of these interventions is challenging (Taylor et al., 2018), in part, due to the requirement for high-quality data from both human cases and from potential sources along the farm-to-fork continuum.

In this study, we review source attribution methodologies within the microbiological approach, which is the most frequently used approach for *Salmonella* (Barco et al., 2013; Mughini-Gras et al., 2018), and their challenges and limitations. We also discuss new challenges raised by recent advances in WGS used in source attribution methods, including the benefits and limitations of incorporating WGS into source attribution models. We focus this review particularly on methods for source attribution relevant to *Salmonella*, including methods used on *Campylobacter*, given that *Campylobacter* is also a foodborne pathogen that can populate different animal reservoirs. These methods could potentially be used with *Salmonella*.

## Microbiological Source Attribution

Attribution of human cases of salmonellosis to sources of transmission and/or infection is essential for the identification of transmission hotspots, the development of control strategies, and the implementation and assessment of interventions. Source attribution methodologies can be performed at three levels in the farm-to-fork continuum: (1) Point of production, that is, animal reservoirs in farms, (2) point of distribution, that is, processing industries and retail; and (3) point of exposure, that is, food preparation and consumption (EFSA, 2008; Ravel et al., 2017).

Most of the previous work on *Salmonella* has focused on the point of production (Barco et al., 2015; David et al., 2013; De Knegt et al., 2015; Hald et al., 2007; Hald et al., 2004; Mullner et al., 2009; Ravel et al., 2017), enabling the assessment of different interventions in the control of pathogens at the reservoir level before reaching other possible transmission routes. Fewer studies have worked with the point of distribution (Guo et al., 2011), the point of exposure (Christidis et al., 2020; Ravel et al., 2017), or utilizing a combination of data from different points (Boysen et al., 2014; Hurst et al., 2023; Mughini-Gras and van Pelt, 2014; Mughini-Gras et al., 2018; Mughini-Gras et al., 2014; Ravel et al., 2017).

Microbiological methodologies of source attribution can be further divided into microbial subtyping methods and comparative exposure assessment. Described in greater detail below, these approaches encompass several methods of source attribution and have both distinct and shared strengths and limitations (Pires et al., 2014).

### Microbial subtyping methods

Microbial subtyping is a technique that allows for differentiation among bacterial isolates (Barco et al., 2013; Wiedmann, 2002). This method compares the set of subtypes of the pathogen in each source with the set of subtypes from human cases. The method relies on accurately matching and distributing the cases of human illness from a particular subtype over the possible sources where that subtype is found.

These so-called “frequency-matching” methods are the most commonly used for foodborne pathogen source attribution (EFSA, 2008; Mughini-Gras et al., 2018). One goal of subtyping is to identify strains that can discriminate among the potential sources or, said differently, identify strains that are source specific. The Kentucky serotype of *Salmonella*, for instance, is almost exclusively found in poultry manure, so any case found with the Kentucky strain is very likely to have originated from this reservoir (Dunn et al., 2022; Murray et al., 2023). A work by Hurst et al. (2023) provides potential metrics to evaluate the subtype definition used in the attribution model. These metrics can aid in achieving optimal balance among source specificity of subtypes, missing data, and level of discrimination power of subtypes.

When using a frequency-matching algorithm, it is important to include all possible sources of transmission of a pathogen so that cases may be matched to their true source (Barco et al., 2013; Mughini-Gras et al., 2019; Pires et al., 2014). Similarly, cases whose subtypes are not found in any of the sources should be discarded in frequency-matching methods. Thus, using a subtyping method that minimizes these unmatched cases is an important consideration (Pires et al., 2014; Pires et al., 2009). Subtypes are defined by either phenotyping methods (e.g., serotyping, phage-type, and antimicrobial resistance) or by genotyping methods (e.g., PFGE and comparative genomic fingerprinting [CGF]) (Barco et al., 2013; Ferrari et al., 2017; Yan et al., 2004). For *Salmonella* isolates, PFGE is the most extensively used method, standardized to support the comparison of isolates between human cases and sources and between and within countries.

#### Frequency-matching methods

The Dutch model and the Hald model, and their modifications, have been extensively used for source attribution of foodborne pathogens (David et al., 2013; De Knegt et al., 2015; Guo et al., 2011; Hald et al., 2007; McLure et al., 2022; Mughini-Gras et al., 2018; Mughini-Gras et al., 2014; Vieira et al., 2016). The Dutch model is a frequentist model that compares the number of human cases of a pathogen subtype *i* with the number of isolates of the same subtype in each source *s* (Hald et al., 2004). The Hald model is based on the Dutch model and relies on estimating the expected number of human cases of subtype *i* from each source *s*, *λ_is_.* To allow for appropriate uncertainty in the parameter estimation process, Bayesian inference is applied to the process (Hald et al., 2004; Mullner et al., 2009), with *λ_is_* equal to

$$\lambda_{is}=p_{is}q_{i}a_{s},$$

where *p_is_* is the proportion of subtype *i* in source *s*, *q_i_* is the subtype-dependent factor that summarizes survivability, virulence, and transmissibility of the pathogen, and *a_s_* is the food source-dependent factor representing the source ability to act as a vehicle for the foodborne pathogen and the differences in monitoring systems of each source. The observed number of human cases of subtype *i*, *o_i_*, follows a Poisson distribution:

$$o_{i}=Poisson\left(\sum_{s}\lambda_{is}\right)$$

*p_is_* is given by the data, where *q_i_* and *a_s_* are parameters of the model.

This results in an overspecified model, given that *T_i_* is the total number of subtypes and *T_s_* is the total number of sources, then there are *T_i_* + *T_s_* parameters, but only *T_i_* independent data points (David et al., 2013; Miller et al., 2017; Mullner et al., 2009). Extra assumptions on *q_i_* and *a_s_* are introduced to reduce the number of parameters, such as *q_i_* is equal for some subtypes and *a_s_* is equal for some food types or sources, yielding *a priori* grouping of subtypes (Hald et al., 2004).

However, no quantification of uncertainty among all possible groupings is available (Miller et al., 2017). The modified Hald model tackles this issue by modeling *q_i_* as random observations from the distribution of characteristics of the pathogen given by $log\left(q_{i}\right)∼N\left(0,\tau \right)$, where τ controls the variation of these characteristics. The following prior distribution is used for $\tau ∼Gamma\left(0.01,0.01\right)$ (Mullner et al., 2009). Moreover, data can be split into different periods to achieve identifiability, given that *q_i_* and *a_s_* are assumed to be constant over time. However, the parameters of this model are still weakly identifiable, resulting in slow convergence of Markov chain Monte Carlo (MCMC) algorithms used to fit the method (David et al., 2013; Miller et al., 2017). MCMC algorithms are used to sample values from a probability distribution (posterior) using prior information and an underlying model (likelihood).

To make the original model more robust, the modified Hald model incorporates uncertainty on the prevalence parameter by defining $p_{is}=\pi_{s}r_{is}$, where π_s_ is the prevalence over all types in source *s* and *r_is_* is the relative occurrence of subtype *i* among the subtyped isolates from source *s*. A beta distribution is used as prior for p_is_ (π_s_∼Beta(1, 1)), and a Dirichlet distribution of size *T_i_* is used as a prior for r_is_ (r_is_∼Dir(1, 1,…,1)) (Mullner et al., 2009).

Recent work by Miller et al. (2017) developed a new method that fits a joint model for both human cases and source samples. This method addresses the weak identifiability issue by using a nonparametric Bayesian clustering method to group the subtypes, thus incorporating uncertainty on all possible groups of subtypes. This approach reduces the number of parameters and does not make any strong assumption on τ.

With the advent of WGS, greater power of discrimination among isolates can be achieved, resulting in higher accuracy for source attribution inference. Moreover, WGS techniques have become more practical and less expensive. Studies have even shown that the benefits of WGS outweigh the cost (Alleweldt et al., 2021; Brown et al., 2021; Glass-Kaastra et al., 2022). As a result, WGS methods are gradually replacing phenotyping and other genotyping methods worldwide.

However, in resource-poor settings, it is still challenging to implement WGS at its full power and with robust sampling (Mather et al., 2015). WGS isolates can be compared across various degrees of similarity, providing different resolutions for genotyping. For example, single nucleotide polymorphism (SNP) analysis compares SNPs across each aligned genome, while core-genome multilocus sequence typing (cgMLST) and whole-genome multilocus sequence typing (wgMLST) use gene-to-gene comparisons. Defining the optimal resolution for genotyping is not clear and may depend on the sources examined (Collineau et al., 2019; Mughini-Gras et al., 2018). Once the sequenced isolates are subtyped, frequency-matching methods can be used. However, WGS data also bring the possibility of new approaches to link sources with cases, as exemplified in the next sections.

#### Population genetic methods

Population genetic methods are a powerful tool with applications to source attribution inference utilizing WGS data. These methods model the organism's evolutionary history and have been extended for source attribution applications for *Salmonella* and *Campylobacter* (Barco et al., 2015; Mughini-Gras et al., 2014; Wilson et al., 2008). One example is the asymmetric island model, which models the DNA sequence evolution and zoonotic transmission of the pathogen (Wilson et al., 2008). Each source is considered a population of pathogens, that is, an island.

Pathogens can migrate among populations and evolve through mutation and recombination. The model estimates migration rate, mutation, and recombination parameters and uses those to assign the probability of each human case isolate having originated from one of the source populations (Wilson et al., 2008). Given that population genetics model the evolution of the pathogen, unique strains (in humans) may be assigned to a source rather than be excluded from the dataset as is necessary for frequency-matching methods.

Another population genetic method for source attribution is STRUCTURE (Jehanne et al., 2020; Mughini-Gras et al., 2021; Mulder et al., 2020; Saif et al., 2022). STRUCTURE uses model-based clustering, which assigns a cluster (population) to each sample, while simultaneously estimating the allele frequency in each population (Pritchard et al., 2000). STRUCTURE assumes that the allele frequency within a population is constant and that the association between different genes is completely random (independent) (Pritchard et al., 2000). In other words, the model associates variability among alleles with population grouping by structuring the samples into clusters.

When extending the application of this model to source attribution, each population (cluster) would be a source, and the human case isolates would be classified among these clusters. The STRUCTURE algorithm can also consider admixture and, as such, the possibility of the introduction of new lineages into a population (or in human cases). The initial algorithm has transformed over the years to address ancestry, dominant marker, and prior information on the groups. In addition, there have been extensions developed to address issues ranging from computational speed to properties of the model, such as considering the spatial distribution of the populations (November, 2016).

However, with the large datasets generated by WGS, the computation time required for these algorithms can be substantial, usually increasing linearly with the number of loci (Pérez-Reche et al., 2020). STRUCTURE, for example, works on short genotypes consisting of, at most, only hundreds of loci, which does not encapsulate all available information of the WGS data.

Many methods have been developed to select markers (features) on the genome that provide higher discrimination among strains and reduce the size of the datasets (Banks et al., 2003; Manel et al., 2002; Pérez-Reche et al., 2020; Storer et al., 2012). These features can be used as input for the source attribution algorithms, resulting in less computational time. Recent work proposed a minimal multilocus distance method to attribute cases to sources, which is fast enough to deal with thousands of loci, while other work suggests a method to select optimal markers from the genotype using information theory (Pérez-Reche et al., 2020).

#### Novel methods of source attribution

Finding hidden complex patterns through Machine Learning (ML) algorithms usually requires a larger amount of data (James et al., 2022). Therefore, ML algorithms are suitable for analyzing WGS data (Lupolova et al., 2019). ML algorithms applied to source attribution can be either unsupervised or supervised learning techniques (Lupolova et al., 2019). For unsupervised learning, there is no label in the data, and the algorithm will group the data into clusters based on their similarities, also known as clustering methods (James et al., 2022). Each cluster can then be associated with a source.

On the other hand, supervised learning uses labeled data to learn hidden characteristics and patterns to categorize the data based on these labels (sources) (James et al., 2022). The model learns using a training dataset and utilizes a testing dataset (data not seen before) for performance evaluation.

Models are often trained on the isolates with known sources of the pathogen. Then, the final model can be used to predict labels (or sources) for unlabeled data. For optimal model hyperparameter tuning and establishing a more robust and unbiased model development process, it is recommended to perform validation procedures, such as cross-validation (James et al., 2022). This involves partitioning the training dataset into distinct subsets, where some are used to train the model and others for model validation (Lupolova et al., 2019). Features or predictors are variables in the input data mapped to the labels through an empirical relationship learned by the model.

There has been an increase in source-attribution studies using ML and classification algorithms (Lupolova et al., 2019; Lupolova et al., 2017; Munck et al., 2020). Recent analyses with *Salmonella* isolates were developed using supervised learning algorithms such as random forests (RF), logit boost, and support vector machines (SVM) for source-attribution problems, and supervised multiclass classification algorithms such as multinomial logistic regression (MLR) (Duarte et al. 2021; Guillier et al., 2020; Lupolova et al., 2017; Munck et al., 2020; Zhang et al., 2019).

Zhang et al. used genomic data of *Salmonella* Typhimurium from different countries to develop a RF algorithm to attribute sources across animal reservoirs to outbreak cases. Their data consisted of genomes from different countries, spanning a significant period (2007–2013, 2015–2017) and focusing on outbreak data. The final dataset comprised 1473 isolates after removing 744 redundant isolates to avoid bias due to sampling similar strains. From these, 1041 genomes were from animals and used to train the classifier. Their final input data comprised 3137 features—1882 core genome SNPs, 150 quality indels, and 1105 source discriminatory accessory genes. The model predicts four animal reservoirs: poultry, wild birds, bovine, and swine, with an accuracy rate of 82.9%.

Because an ML classifier is restricted to the classes represented in the training data, Zhang et al. added a tool to further classify its prediction as precise or imprecise. Thus, isolates from sources not present in the training data are classified as one of the sources included in the model, but its prediction may be identified as imprecise. In addition, they build an extra RF classifier with humans as a source to compare their results with Lupolova et al.'s (2017) work (Wheeler, 2019; Zhang et al., 2019).

They found that only 36.96% of human cases were assigned to humans, as opposed to 90% of Lupolova et al.'s studies. This was due to Lupolova et al. having closely related human isolates (around 85% shared their most recent common ancestor) in their training dataset, resulting in the high accuracy of human host prediction. Given Zhang et al. removed redundant isolates, their data have only 36.9% of human isolates sharing the same most recent common ancestor.

Lupolova et al. used SVM to predict the isolation host for each genome and analyze host specificity. They want to determine whether genetic content can discriminate among interspecies transmission. Their genome data span an extended range of years (1945–2016) and countries and contain different serovars: *Salmonella* Typhimurium (human, bovine, swine, and poultry), *Salmonella* Typhi (human), and *Salmonella* Dublin (human and bovine). They build an SVM model for each host with final input data of protein variants as features and hosts as labels. Thus, an isolate could be assigned for multiple sources—making it a generalist strain.

However, the majority (94%) was assigned to only one host. The model misclassified some isolates; this could be because the data do not incorporate all the genetic features of a source or the strain is transient between hosts. The final model predictions were highly accurate (ranging from 67% to 90%) (Lupolova et al., 2017). These two studies highlight the importance of model building, feature selection, and appropriate data processing when dealing with ML models and WGS data. It is possible to achieve different conclusions by following other procedures.

Recent work by Munck et al. developed a boosting algorithm (logit boost) to classify sporadic human cases of *Salmonella* Typhimurium in Denmark among the following sources: Broilers, layers, cattle (domestic), cattle (import), ducks (import), pigs (domestic), and pigs (import). They used human, food, and animal isolates collected from an integrated surveillance system in Denmark over 2 years to ensure the data represent all true sources. Their input data consisted of cgMLST, which was further reduced to only 17 loci using feature selection techniques.

All sources' isolates were correctly predicted, except for 38% of domestic pigs and 27% of imported pigs, which were wrong classified as poultry. Their final model accuracy was 92%. Of all human sporadic cases, 81% were attributed. The human cases not attributed were either infected from a source not in the training dataset or a strain not captured in the training data. They compared their model against the Bayesian Hald model (Hald et al., 2004) in the same dataset. The input data for the Hald model were the isolates' multilocus variable-number tandem-repeat analysis (MLVA) profile and resistance profile. The results were similar, but only 49% of human cases were attributed (Munck et al., 2020). Both models draw similar conclusions regarding the sources, corroborating ML as a new, robust, and efficient tool for source attribution.

Guillier et al. developed an MLR, an extension of logistic regression to allow multiclass classification to predict the source of environmental strains of *Salmonella* Typhimurium and its monophasic variant. Ninety-eight bacterial isolates were collected from 2010 to 2015; 69 were from animals (pigs, poultry, and ruminants) and 19 were from the environment (no source). They first calculated the accessory genes (noncore genome) enriched in each source to use as input data in the MLR. Then, they use Aikake information criteria to decide which accessory genes to include as features in the final model (eight genes). The chosen model had an accuracy of 74% (Guillier et al., 2020). Table 1 summarizes the main properties of each model.

**Table 1.** Summary of Properties of Each Machine Learning Source Attribution Model Using Whole-Genome Sequencing: Base Model, Data Collection, Input Features, Sources (Labels), Percentage of Attributed Human Cases, Comparison with Other Models (if Existent), and *Salmonella* Serotype

Author	Munck et al.	Zhang et al.	Lupolova et al.	Guillier et al.
Models	Logit Boost	Random Forest	Support Vector Machines	Multinomial logistic regression
Data collection	Human, food, and animal isolates collected from integrated surveillance system in Denmark	Outbreak data on human, food, environment, wild and livestock animal from different countries	Human and animal isolates over many countries and ranging from 1945 to 2016.	98 Bacterial isolates collected over 2010–2015 (19 without label)
Input Features	cgMLST (17 loci after feature reduction)	Core genome SNPs, high quality indels, and source discriminatory accessory genes	Protein variants (up to 1000–1500)	Eight accessory genes (noncore genome)
Sources	Broilers, layers, cattle, cattle (import), ducks (import), pigs, pigs (import)	Bovine, swine, wild bird, and poultry	Isolation host: avian, bovine, human, swine	Pigs, poultry, and ruminants
Attribution of cases	81%	42%	Not applicable	25 Out of 29 environmental strains
Accuracy	92%	83%	67–90%	74%
Model comparison	Hald model using MLVA profile and resistance profile: fit of 0.9 and 49% attribution of human cases	Not applicable	Not applicable	Not applicable
Serovar	*Salmonella* Typhimurium	*Salmonella* Typhimurium	Multiple serovars (Typhi, Dublin, Typhimurium)	*Salmonella enterica* Typhimurium and *Salmonella enterica* 1,4,[5],12:i:-

cgMLST, core-genome multilocus sequence typing; MLVA, multilocus variable-number tandem-repeat analysis; SNP, single nucleotide polymorphism.

A study compared three ML source attribution models, SVM, RF, and neural networks (NN), on the same dataset of *Salmonella* Typhimurium. All models arrived at similar results with similar accuracy (75–90%). RF is the most user-friendly, can predict multiple classes at once, and provides a list of the most relevant features. NN is highly scalable and can predict various classes; however, it requires technical knowledge. In summary, any of these models effectively attributes the source for *Salmonella* Typhimurium (Lupolova et al., 2019).

It is possible to expand source attribution even further. Recent work developed a source attribution model based on hierarchical clustering to rapidly identify and trace salmonellosis' geographical sources, rather than points in the food chain, from WGS data (Bayliss et al., 2023).

It is noteworthy to mention the work by Arning et al. (2021), which provides a comparison of performances of different ML algorithms for attributing sources of campylobacteriosis cases. In summary, they identified the best-performing ML algorithms for different resolutions of sequence data: multilocus sequence typing (MLST), cgMLST, and WGS. They tested 14 supervised learning algorithms, ranging from simple learners such as K-nearest neighbors, decision tree-based algorithms to deep learning algorithms such as NN, and the asymmetric island model, iSource. They found ML outperforms iSource.

In addition, some studies have applied weighted network analyses, a clustering method, to perform source attribution. In this methodology, each node in the network is an isolate, and links between isolates represent their genetic distance. Isolates from the same sources would then be clustered together. It has been found that this method remains robust independent of the resolution of WGS data used, whether SNP, cgMLST, or wgMLST (Merlotti et al., 2020; Wainaina et al., 2022).

There are still challenges to applying source attribution to WGS data. Following, we cover some of them:

(1) A well-known issue is unique strains in unlabelled data. For Bayesian models, subtypes not included in the sources can be removed. ML models can only classify strains that are in the training data so predictions can further be labeled as precise or imprecise to ensure accurate classification (Munck et al., 2020; Zhang et al., 2019). Unique isolates may be assigned to an existent source for population genetics, given that pathogen evolution is considered in the model.
(2) Another common issue is when some sources are poorly sampled, which could generate incorrect predictions. A potential solution is to upsample or downsample the dataset (Lupolova et al., 2019; Munck et al., 2020). Moreover, for WGS data, it is essential to remove redundant genomes by analyzing some genetic features, such as the number of SNPs separating each isolate (Zhang et al., 2019), to avoid overinflating model accuracy (such as similar strains from outbreak data in a population-level study).
(3) Predictions need to be adjusted for “unknown sources” not included in the data, for example, by allowing the classification into an extra source using isolates from other sources (environment) to inform the “unknown source” or adding a tool to identify imprecise predictions (Zhang et al., 2019).

All the above highlight the need for having a robust sampling process of the true sources.

### Comparative exposure assessment

Comparative exposure assessment is a microbiological methodology that focuses on the point of exposure and transmission routes rather than animal reservoirs. There have been studies that apply comparative exposure assessment to attribute sources of exposure for some foodborne pathogens such as *Salmonella* (Christidis et al., 2020; Fajardo-Guerrero et al., 2020) and *Campylobacter* (Evers et al., 2008; Pintar et al., 2017). The comparative exposure assessment estimates the average number of pathogens that individuals in a population are exposed to in each source and route per day.

The exposure, E, is defined as the average number of organisms that individuals are exposed to in a day (units of cells/person/day) for a pathway of a specific source. It can be formulated as following:

E=f×i×p×c,

where *f* is the frequency of ingestion events (events/day), *i* is the total mass (or volume) consumed per individual per event (mass/event/person), *p* is the probability that the ingested item is contaminated with the pathogen, and *c* is the concentration of pathogen cells per mass (volume) in the ingested item, given it is contaminated (cells/mass) (Christidis et al., 2020).

Exposure is estimated separately for all relevant transmission routes within the categories of food, animal contact, and environment (EFSA, 2008). For each transmission route, adaptations to the calculation of each component of the exposure equation may need to be implemented. For example, for food contamination, an extra term indicating raw, undercooked, and cooked consumption may be included. Comparative exposure assessment estimates the relative contribution of each transmission route to the population's total exposure, which is directly related to the likely sources of cases of human illness (Pintar et al., 2017; Ravel et al., 2017). In this way, one can assess which sources, transmission pathways, and points along the pathway have a larger risk for the population and implement interventions to decrease this risk.

However, the possibility of cross-contamination and different transmission routes make directly linking the point of exposure to animal reservoirs more difficult. There are many techniques to achieve this linkage or provide a better understanding of possible routes. For instance, a meta-analysis combined results from attribution studies across reservoirs and transmission routes and estimated attribution proportions for the transmission pathways (Mughini-Gras et al., 2022). A more precise estimate is possible by either combining frequency-matching methods with comparative exposure assessment or case–control studies, allowing for the control of exposure when estimating the frequency of cases in each source.

This generates a complete picture of the transmission pathways from the point of production to exposure, which can better inform risk management in the prioritization of control strategies for each transmission route (EFSA, 2008; Hurst et al., 2023; Mughini-Gras and van Pelt, 2014; Mughini-Gras et al., 2019; Mughini-Gras et al., 2018; Mughini-Gras et al., 2014; Ravel et al., 2017). A study by Mughini-Gras et al. combined multiple microbial subtyping frequency-matching methods with a comparative exposure assessment to estimate the contribution of each point of exposure to salmonellosis. They incorporated an exposure term in the calculation of *λ_is_*. For the Hald model, they had,

$$\lambda_{is}=m_{s}c_{s}p_{is}q_{i}a_{s},$$

where *m_s_* represents the consumption of source *s*, *c_s_* is the probability of the source being eaten raw/undercooked, *p_is_* is the proportion of subtype *i* in source *s*, *q_i_* is the subtype-dependent factor, and *a_s_* is the food source-dependent factor, as previously defined. For the Dutch model, controlling the consumption of the source without considering the probability of eating raw/undercooked food led to pig as the highest contributing source and table eggs as second, which is inconsistent with common knowledge in *Salmonella* epidemiology. Thus, it is necessary to consider the consumption weight and the likelihood of the food being undercooked to properly estimate the contribution of each source when using the Dutch model. The Hald model grants expected results regardless of the inclusion of food consumption data.

### Impact on Salmonellosis

In the case of *Salmonella*, efforts to reduce incidence have shown positive results. In 2019, it was estimated that illnesses caused by *Salmonella* in Canada decreased by more than 25,000 cases relative to the previous 5 years (Glass-Kaastra et al., 2022). Successful source attribution through genomic-based surveillance contributed to the implementation of new effective, targeted interventions, driving the reduction of cases (Glass-Kaastra et al., 2022; Morton et al., 2019). WGS source attribution implementation generated a more accurate and specific linkage to products, providing the evidence needed for new control requirements (Morton et al., 2019). The implementation of WGS in the United States has prevented around 25,000 cases of foodborne illness, saving around 500 million U.S. dollars (Brown et al., 2021; Glass-Kaastra et al., 2022). Work on case studies on WGS implementation across Europe and America found that the benefits of WGS outweigh the cost (Alleweldt et al., 2021).

In brief, the implementation of WGS provides better accuracy, more specificity on outbreak linkages, generate better evidence to inform control policy, and improve understanding of disease transmission. Recent work by Hurst et al. (2023) shows a decline in the percentage of cases attributed to chicken breasts by one-third from 2015 to 2019 and in the incidence rate of salmonellosis by one-third in the same period in Canada. However, despite the observed reduction in cases, the incidence of *Salmonella* infections remains high, with an estimated 70,833 cases of illness in Canada in 2019 (Glass-Kaastra et al., 2022), highlighting that further efforts are needed.

## Conclusion

Source attribution methods have been extensively applied to identify transmission routes and animal reservoirs of foodborne pathogens such as *Salmonella*. Frequency-matching approaches have been widely utilized for microbial subtyped data to estimate the probability of a human case originating from an animal reservoir. The growth of WGS and its popularization for source attribution studies has increased the development and application of novel methods. WGS provides high-resolution power to discriminate isolates, which can increase the accuracy of frequency-matching approaches. Evolutionary and population genetics algorithms may also be used to link sources to human case isolates. The large size of WGS data further allows for introducing ML classification methods in source attribution, where each source is a class. This work summarizes well-known source attribution methods and novel methods.

However, there are still challenges to overcome, such as the computational efficiency of these methods, given the large data size. To address this issue, one may select a few features (genetic markers) with high discrimination power among sources and reduce the input data size. Another well-known problem is that available data often lack complete information about the various sources, which highlights the importance of having a solid and integrated surveillance system encompassing all one health spheres—animal, human, and environmental (Mather et al., 2015; Mughini-Gras et al., 2019; Pires et al., 2014). The field of source attribution is still evolving, with new methods arising, which improve on the older ones. Furthermore, the richness of WGS data has not yet been fully utilized, although progress is being made.

Including WGS data in source attribution can provide better evidence to inform policy development and prioritize intervention strategies to control salmonellosis. In addition, they help better understand the complex interactions of pathogens with animals, humans, and the environment, such as determining genetic features responsible for host specificity and adaptability and geographical distribution of *Salmonella*. Therefore, continued improvement, development, and generalization of source attribution methods are essential to advance our understanding and control of *Salmonella* transmission.
